# Serotonin-Derived Fluorophore: A Novel Fluorescent Biomaterial for Copper Detection in Urine

**DOI:** 10.3390/s23063030

**Published:** 2023-03-10

**Authors:** Mariagrazia Lettieri, Simona Scarano, Laura Caponi, Andrea Bertolini, Alessandro Saba, Pasquale Palladino, Maria Minunni

**Affiliations:** 1 Department of Chemistry ‘Ugo Schiff’, University of Florence, 50019 Sesto Fiorentino, Italy; 2 Laboratory of Clinical Pathology, University Hospital of Pisa, 56126 Pisa, Italy; 3 Department of Surgical, Medical and Molecular Pathology and Critical Care Medicine, University of Pisa, 56126 Pisa, Italy

**Keywords:** serotonin, serotonin-derived fluorophore, urine analysis, fluorescence quenching, copper detection, copper-dependent diseases, copper poisoning

## Abstract

We took advantage of the fluorescent features of a serotonin-derived fluorophore to develop a simple and low-cost assay for copper in urine. The quenching-based fluorescence assay linearly responds within the concentration range of clinical interest in buffer and in artificial urine, showing very good reproducibility (CV_av_% = 4% and 3%) and low detection limits (16 ± 1 μg L^−1^ and 23 ± 1 μg L^−1^). The Cu^2+^ content was also estimated in human urine samples, showing excellent analytical performances (CV_av_% = 1%), with a limit of detection of 59 ± 3 μg L^−1^ and a limit of quantification of 97 ± 11 μg L^−1^, which are below the reference value for a pathological Cu^2+^ concentration. The assay was successfully validated through mass spectrometry measurements. To the best of our knowledge, this is the first example of copper ion detection exploiting the fluorescence quenching of a biopolymer, offering a potential diagnostic tool for copper-dependent diseases.

## 1. Introduction

**Scheme 1:**
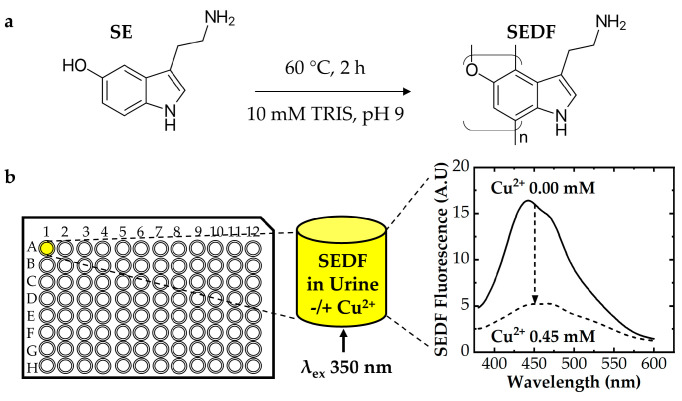
(**a**) Putative SEDF structure obtained by SE monomer polymerization upon linkage through the benzene ring after 2 h at 60 °C in 10 mM TRIS buffer pH 9; (**b**) Cu^2+^ detection in a 96−well microplate by reading the SEDF fluorescence quenching upon a [Cu^2+^] increase in urine.

Copper ion is essential to living systems, regulating many physiological functions, acting as a cofactor of numerous enzymes (e.g., dopamine β-hydroxylase, tyrosinase, and cytochrome c oxidase [1]), contributing to cellular and tissue growth, and working as an antioxidant [2]. The World Health Organization (WHO) recommends an intake of 30 µg per kilogram of body weight per day [3]. However, an excess of this heavy metal leads to protein structure modification, interfering with the exchange of zinc in metalloproteins and compromising most cellular functions [4]. Recently, it was demonstrated that an accumulation of Cu^2+^ affects the activity of dopaminergic neurotransmitters, potentially leading to neurodegenerative disorders and mental issues, including anxiety, depression, language, and cognitive impairment [5,6,7]. In this context, a few fluorescent methods were developed for Cu^2+^ quantitative analysis in urine [8,9,10,11,12,13,14]. In this study, for the first time, the fluorescent properties of a novel fluorophore derived from serotonin (SE) self-oxidation and polymerization were exploited for sensitive Cu^2+^ detection in human urine without matrix pretreatment. SE is a neurotransmitter involved in multiple important physiological processes, such as memory, learning, anxiety, depression, cognition, vomiting, and vasoconstriction [15]. Very few studies describe the formation of SE oligomers [16,17,18,19], including polyserotonin (PSE) obtained using horseradish peroxidase (HRP) [19], and serotonin-based nanoparticles (PSE-NPs) [16,17] as multifunctional material for free radical scavenging, bioelectrical, and biomedical applications. In this paper, the highly emitting SE-derivative fluorophore (SEDF) was obtained by heating the SE monomer at an alkaline pH (Scheme 1a). SEDF was characterized by means of UV-Vis spectroscopy and mass spectrometry. The fluorescent properties of SEDF were outstanding when compared with the fluorescent properties of polymers obtained using other endogenous neurotransmitters, i.e., polydopamine (PDA) and polynorepinephrine (PNE) [20,21]. The analytical performances of the fluorescence detection strategy were firstly evaluated by determining the Cu^2+^ in buffer and in artificial urine. Then, based on these results, we proceeded to determine the Cu^2+^ concentration in human urine samples (Scheme 1b) observing the reference values sufficient to detect the early stage of diseases associated with Cu^2+^ accumulation, such as chronic liver disease, acute hepatitis, and, primarily, Wilson’s disease (WD), a rare inherited disorder that can lead to excess storage of copper in the liver, brain, and other organs [22,23]. Patients who suffer from WD have high urinary copper values between ca. 200 and 400 μg per day [24,25]. Values of Cu^2+^ higher than 100 µg per day are strongly indicative of this disease [23]. Common diagnostic assays for WD are based on the ceruloplasmin (Cp) concentration and on hepatic and urinary Cu^2+^ detection [23,26]. However, the Cp test is inaccurate for the estimation of free Cu^2+^, whereas the evaluation of hepatic copper requires an invasive procedure and, often, the heterogeneous Cu^2+^ distribution within the liver leads to false negative results. Differently, the experimental procedure here developed for copper estimation would be noninvasive, fast, very simple, green, and low cost if compared to the reference instrumental approach for copper quantification in urine samples, such as inductively coupled plasma mass spectrometry (ICP-MS) [27], here used to validate the fluorescence method. This work aims to propose a new diagnostic tool for copper-dependent diseases, representing, to the best of our knowledge, the first example of copper ions detection exploiting the fluorescence quenching of a biopolymer.

## 2. Materials and Methods

### 2.1. Chemicals

Serotonin (C_10_H_12_N_2_O, 98% pure), tris(hydroxymethyl)aminomethane (C_4_H_11_NO_3_, 99% pure), sodium hydroxide (NaOH, ≥99% pure), hydrochloric acid (HCl, 37% *w*/*w*, ≥99% pure), sodium chloride (NaCl, ≥99% pure), potassium chloride (KCl, ≥99% pure), lithium chloride (LiCl, 99% pure), and magnesium chloride (MgCl_2_, 98% pure) were obtained from Thermo Fisher Scientific (Parma, Italy). Barium chloride (BaCl_2_, ≥99% pure), copper sulfate (CuSO_4_, ≥99% pure), bismuth (III) nitrate (Bi(NO_3_)_3_·5H_2_O, 98% pure), aluminum chloride (AlCl_3_, 98% pure), zinc (II) sulphate (ZnSO_4_·7H_2_O, 98% pure), cadmium (II) sulfate (CdSO_4_·8H_2_O, ≥99% pure), mercury (II) chloride (HgCl_2_, 98% pure), silver chloride (AgCl, 99% pure), gold (III) chloride (HAuCl_4_·3H_2_O, 99% pure), nickel (II) chloride (NiCl_2_·6H_2_O, 99% pure), chloroplatinic acid solution (H_2_PtCl_6_, 99% pure), palladium (II) oxide (PdO, 99% pure), iron (III) chloride (FeCl_3_ × 6H_2_O, 97% pure), potassium permanganate (KMnO_4_, 98% pure), potassium chromate (K_2_Cr_2_O_7_, ≥99% pure), cobalt (II) chloride (CoCl_2_·6H_2_O, 98% pure), ruthenium (IV) oxide (RuO_2_, ≥99% pure), acetonitrile (C_2_H_3_N, ≥99% pure), DHB (2,5-dihydroxy benzoic acid, C_7_H_6_O_4_, 98% pure), glutamic acid (1 μM, C_5_H_9_NO_4_, 99% pure), glucose (2 μM, C_6_H_12_O_6_, 99% pure), ascorbic acid (2 μM,C_6_H_8_O_6_, ≥99% pure), hypoxanthine (9 μM, C_5_H_4_N_4_O, ≥99% pure), uric acid (4.5 μM, C_5_H_4_N_4_O_3_, ≥99% pure), creatinine (4 μM, C_4_H_7_N_3_O, ≥99% pure), urea (0.5 M, CH_4_N_2_O, ≥99% pure), sodium citrate (2 μM, Na_3_C_6_H_5_O_7_, ≥99% pure), trifluoroacetic acid (TFA, C_2_HF_3_O_2_, 98% pure), quinine sulfate (C_20_H_26_N_2_O_6_S, 99% pure, C_40_H_50_N_4_O_8_S), sulfuric acid (H2SO4 ≥ 98% pure), 1-butanol (C_4_H_10_, HPLC purity reagent grade), water (H_2_O, HPLC purity reagent grade), Triton™ X100 (C_14_H_22_O(C_2_H_4_O)_n_, 98% purity reagent grade BioXtra), and tetramethylammonium hydroxide solution (TMAH, 25 wt% in H_2_O, 99% pure) were purchased from Merck (Milan, Italy). The artificial urine (AU) was from LCTech GmbH (Obertaufkirchen, Germany).

Human urine samples were collected from those destined for destruction at the Clinical Pathology Laboratory of the University Hospital in Pisa. Spiked samples were obtained and tested. Fluorescence measurements and ICP-MS experiments on the urine samples were performed in the Clinical Pathology Laboratory of the University Hospital in Pisa.

### 2.2. Instrumentation

The temperature-controlled synthesis reaction of SEDF was performed using a Thermomixer comfort (Eppendorf, VWR International, Milan, Italy). The fluorescence experiments were performed with fluorimeter FP-6500 (Jasco, Easton, Pennsylvania, USA) using an excitation and emission wavelength of λ_ex_ = 350 nm and λ_em_ = 450 nm, respectively (emission bandwidth: 10 nm; excitation bandwidth: 5 nm; data pitch: 1 nm; scanning speed: 100 nm min^−1^; sensitivity: low) and microplate readers Fluoroskan Ascent (Thermo Fisher Scientific, Milan, Italy) by selecting as filter pairs λ_ex_ = 390 nm and λ_em_ = 460 nm, corresponding to the excitation and emission wavelengths, respectively. The absorbance measurements were obtained using a SPECTROstar Nano UV-Visible Spectrophotometer (Ortenberg, Germany) in 1.0 cm quartz cells at 20 °C. The mass spectrometry measurements, acquired in the positive-ion mode, were performed with a MALDI-TOF/TOF Ultraflex III (Bruker Daltonics, Milan, Italy) (matrix-assisted laser desorption/ionization time-of-flight mass spectrometry) by mixing the samples in a 1:1 ratio with 20 ng μL^−1^ DHB (2,5-dihydroxy benzoic acid) as matrix dissolved in 70% of acetonitrile, 25% trifluoroacetic acid, and 5% ethanol. The mass spectra were acquired in the positive-ion mode.

### 2.3. Assay Protocol

#### 2.3.1. Synthesis of Serotonin-Derived Fluorophore

A total of 2 g L^−1^ of serotonin solution was dissolved in a 10 mM TRIS buffer pH 9.00 and heated at 60 °C for 2 h. Subsequently, the sample was left for 10 min at room temperature, then centrifuged 2 times for 10 min at 10,000 rpm. Finally, the supernatant was collected and stored at 4 °C after the addition of 5 mM HCl. 

#### 2.3.2. Quantum Yield Calculation

The fluorescence quantum yield (*Q*) of the SEDF was estimated using quinine sulfate dissolved in 0.1 M H_2_SO_4_ as the fluorescence reference standard of the known quantum yield (*Q_R_*), as reported by Lakowicz [28]. The quantum yield of the SEDF was calculated using the following equation:

*Q* = *Q_R_* × *I*/*I_R_* × *OD_R_*/*OD* × *n*^2^/*n*^2^*_R_*

where *I* is the integrated fluorescence intensity, *OD* is the optical density at the excitation wavelength, and *n* is the refractive index of the solvent. The subscript *R* indicates the same parameters for the reference fluorophore, in this case quinine sulfate. The absorbances at the wavelength of excitation (λ_ex_ = 350 nm) were kept at A = 0.05 to avoid the inner filter effect [28], thus ensuring a linear dependence of the fluorescence signals to the sample concentration.

#### 2.3.3. Copper Determination via the Fluorescence Quenching-Based Method

Stock Cu^2+^ solutions were prepared in 10 mM Tris pH 9.00 and in artificial urine. The different volumes of copper solution were mixed with solutions of previously synthesized SEDF (see above), immediately recording the fluorescence signal. The decrease in the fluorescence in dependence on the copper concentration was expressed as F_0_/F, where F_0_ is the fluorescence of the SEDF without any metal addition, and F is the fluorescence recorded after the copper addition in buffer, artificial urine, and real urine samples. All measurements were performed at 25 °C in quadruplicates, at least. The limit of detection (LOD) and limit of quantification (LOQ) were calculated by applying the following equations: LOD = 3 × SD_blank_/slope and LOQ = 10 × SD_blank_/slope, where the standard deviation of the blanks (SD_blank_) was divided by the slope of the relative calibration plots.

#### 2.3.4. ICP-MS Measurements

The method here developed was validated using an Agilent Technologies 7900 ICP-MS (Santa Clara, CA, USA) equipped with an ASX-500 Series autosampler and a peristaltic pump for the sample injection. The analyses were performed with the following acquisitions parameters: carrier gas flow rate of 0.80 L min^−1^ (Ar), aerosol dilution flow rate of 0.50 L min^−1^ (Ar), plasma gas flow rate of 15 L min^−1^ (Ar), collision gas flow rate of 4.3 × 10^−3^ L min^−1^ (He), RF power of 1550 W, stabilization time of 20 s, peak pattern of 3 point, 3 replicates, 100 sweeps per replicate, and a peristaltic pump speed of 0.1 rps. The system control and data acquisition and processing were carried out using the ICP-MS MassHunter Workstation^®^ software, version 5.1. For the pre-analytical procedure, the Agilent environmental calibration standard mix, including copper (^63^Cu) at a concentration of 10,000 µg L^−1^, was diluted in deionized water to prepare an initial calibration curve ranging from 0 to 2500 µg L^−1^. The calibration points at 0, 0.1, 0.2, 0.5, 1, 2, 5, 10, 20, 50, 100, 200, and 250 µg L^−1^ were obtained by dilution with water:1-butanol = 98.5:1.5 (*v*/*v*) added with Triton X100, 10 µL L^−1^, and Tetramethylammonium hydroxide solution (TMAH 25% in H_2_O), 100 µL L^−1^. Each calibration point was added with the appropriate amount of germanium (^72^Ge) as the internal standard. Two quality control points were added to attest the accuracy of the analysis. The urine samples, previously centrifuged at 1230 rpm for 15 min to remove the sediment, were analyzed after a dilution of 1:100 with water: 1-butanol solution with the appropriate amount of the internal standard.

## 3. Results and Discussion

### 3.1. Synthesis and Characterization of Serotonin-Derived Fluorophore (SEDF)

**Figure 1:**
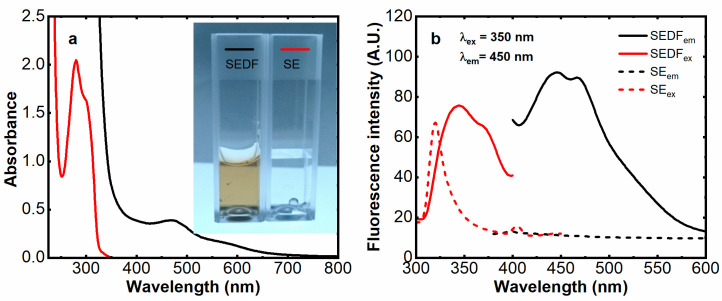
(**a**) UV-Vis spectra of the SE monomer (red line, right cuvette) and synthetic SEDF (black line, left cuvette) in 10 mM TRIS pH 9.00; (**b**) fluorescence excitation (red, λ_em_ = 450 nm) and emission (black, λ_ex_ = 350 nm) spectra of serotonin-derivative fluorophore (SEDF, thick line) and serotonin (SE, dashed line).

Recently, Jeon et al. reported the synthesis of insoluble polyserotonin nanoparticles from the self-oxidation of the SE monomer heated for 2 h at 60 °C in TRIS buffer at pH 9.00 [16]. Repeating the same procedure, we collected the supernatant solution instead of the precipitated nanoparticles, thus obtaining the serotonin-derived fluorophore (SEDF). The SEDF solution was brown in color with a λ_max_ = 470 nm, indicative of a larger conjugated system with respect to the serotonin monomer showing the spectrum typical of a colorless indole derivative with maximum absorbance values at 280 nm and 300 nm (Figure 1a). Analogously, the fluorescence spectra of the SEDF showed a large emission band of approximately 450 nm, whereas in the same conditions the SE monomer was not fluorescent (Figure 1b).

**Figure 2:**
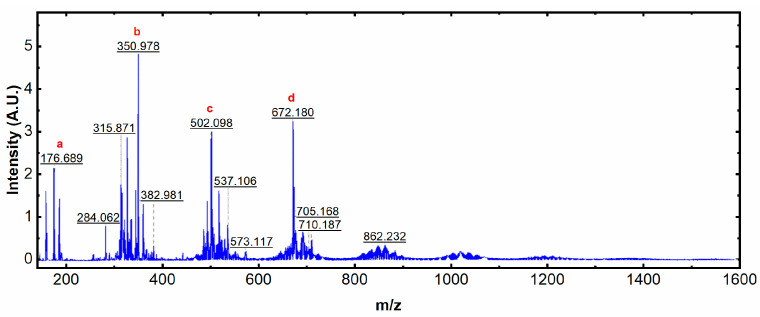
MALDI-TOF/TOF mass spectrum of the SE-derivative fluorophore (SEDF) obtained by heating 2 g L^−1^ serotonin at 60 °C for 2 h in 10 mM TRIS at pH 9.00. The *m*/*z* values compatible with SE monomer (**a**), dimer (**b**), trimer (**c**), and tetramer (**d**) molecules are reported.

Previous works refer to an SE dimer [29,30], while others describe an oligomer obtained through the electrochemical oxidation of serotonin [31,32,33]. To better understand the nature of the serotonin derivative obtained here, we performed mass spectrometry experiments by MALDI-TOF/TOF instrumentation. The peaks highlighted in Figure 2 (*m*/*z* 176.689, 350.978, 502.098, and 672.180) are compatible with monomeric, dimeric, trimeric, and tetrameric structures, possibly obtained upon the linkage through the benzene ring (Scheme 1) [19]. However, the determination of the SEDF structure is out of the scope of this study and requires a much deeper analysis.

**Figure 3:**
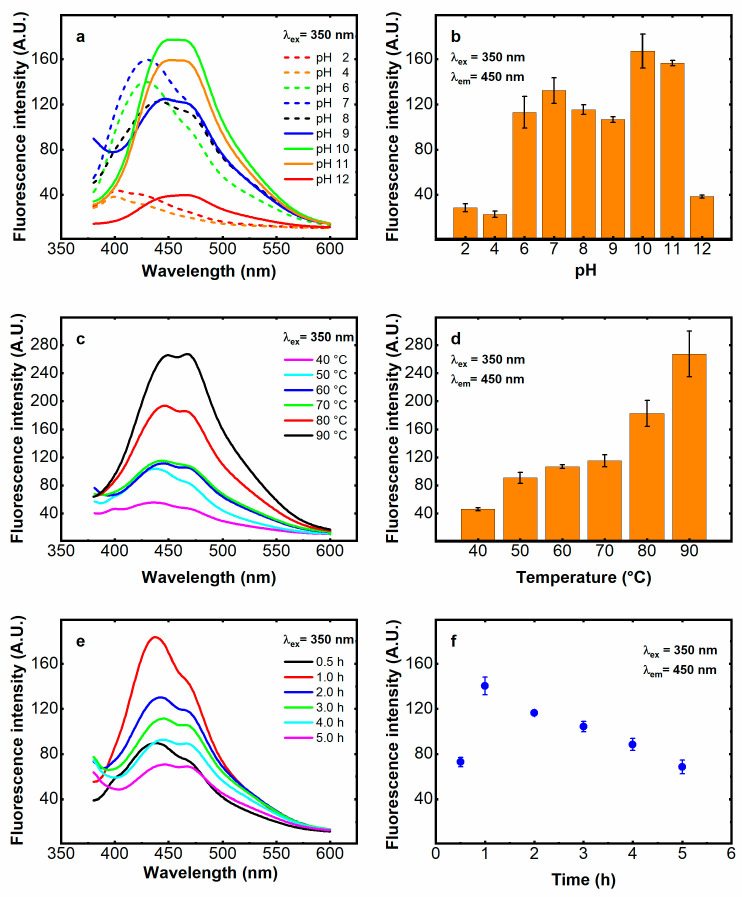
Fluorescence emission (λ_ex_ = 350 nm, λ_em_ = 450 nm) of serotonin-derivative fluorophore (SEDF) obtained by heating 2.0 g L^−1^ SE monomer. The influence of pH 2–12 (**a**,**b**), temperature 40 °C–90 °C (**c**,**d**), and reaction time 0.5–5 h (**e**,**f**) on the fluorescence emission spectra (left) and intensity at 450 nm (right). The error bars represent the standard deviation (n = 4).

The influence of the synthetic parameters, such as pH, reaction time, and starting monomer concentration, on the fluorescent properties of the SEDF were tested (Figure 3) and, notably, the best fluorescence performances of the supernatant coincide with the best conditions for the size-controlled synthesis of polyserotonin nanoparticles [16].

In detail, the influence of the pH on the fluorescence of the SEDF was evaluated between 2.00 and 12.00. The solutions of the SEDF when excited at 350 nm showed emission spectra with a λ_max_ of approximately 400 nm at pH 2.00–4.00, 425 nm at pH 6.00–7.00, and 450–475 nm at pH 8.00–12.00 (Figure 3a,b). The intensity of the fluorescence emission was strongly reduced at a pH below 6.00 or above 11.00. Moreover, highly basic conditions led to heterogeneous mixtures with large scattering phenomena (data not shown). As shown in Figure 3b, the buffer at pH 9.00 resulted in the best fluorescence reproducibility with good fluorescence intensity, and this condition was used for the subsequent studies.

The influence of the temperature on the fluorescence of the SEDF at pH 9.00 for 2 h was evaluated between 40 °C and 90 °C (Figure 3c,d). The fluorescence emission of the SEDF increased with the temperature, and 60 °C resulted in the temperature of choice, representing a good compromise in terms of the signal intensity and reproducibility (Figure 3d).

The influence of the reaction time on the fluorescence of the SEDF was evaluated between 30 min and 5 h at pH 9.00 and 60 °C. The fluorescent spectrum of the SEDF was already visible after 30 min of reaction, reaching the maximum intensity after 1 h (Figure 3e,f). However, the best reproducibility was achieved after 2 h of reaction (Figure 3f).

**Figure 4:**
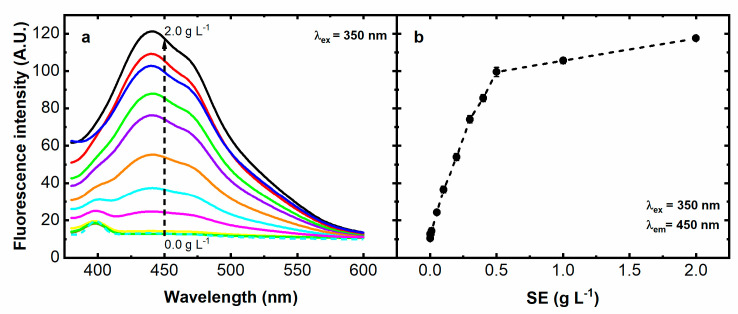
Influence of the serotonin monomer concentrations (0, 0.001, 0.005, 0.010, 0.050, 0.100, 0.200, 0.300, 0.400, 0.500, 1.000, and 2.000 g L^−1^) on the emission signal of the serotonin−derivative fluorophore synthesized after 2 h at 60 °C in alkaline conditions (pH 9.00). The fluorescence emission spectra upon excitation at 350 nm (**a**) and corresponding fluorescence emission intensity at 450 nm (**b**). The error bars represent the standard deviation (n = 4).

Finally, the influence of SE monomer concentration on the fluorescence of SEDF after 2 h at pH 9.00 and 60 °C (Figure 4a,b) was investigated. The largest fluorescence increase was obtained for SE up to 0.5 g L^−1^, reaching a plateau at 2 g L^−1^ SE, which was fixed as the monomer concentration for the following experiments. The synthetic product was stabilized by adding 5 mM of HCl that stopped the oxidation process. The fluorescence intensity in buffer was stable up to 4 h, decreasing to 75% of the starting value after one month (Figure S1).

The fluorescence quantum yield (Q) of the SEDF was estimated in water, as reported in the assay protocol, and the obtained value of 0.025 was higher than the Q reported for the PSE obtained enzymatically (0.017) [19] and here calculated also for the PDA (0.019) and PNE (0.008), indicating the good fluorescent properties of such conjugated serotonin.

### 3.2. SEDF Fluorescence Quenching by Metal Ions

**Figure 5:**
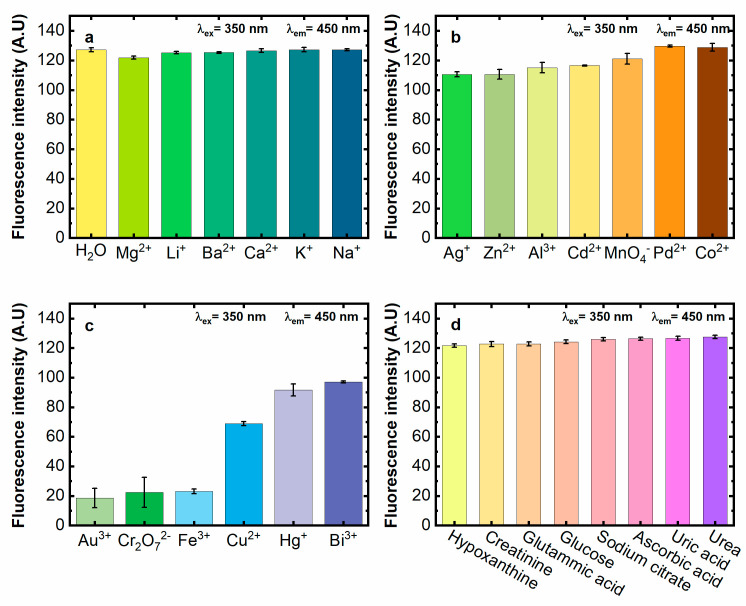
Fluorescence of the SEDF in 10 mM TRIS at pH 9.00 after the addition of 0.5 mM alkaline and alkaline earth metals (**a**), transition metals (**b**,**c**), and organic compounds occurring in urine (**d**). The error bars represent the standard deviation (n = 4).

The effect of several solutions (0.5 mM) of alkaline, alkaline earth (Figure 5a), and transition metals (Figure 5b,c) on the SEDF fluorescence was evaluated, and it was found that only the latter quenched the fluorescence of the SEDF, in particular, Au^3+^, Cr_2_O_7_^2−^, Fe^3+^, and Cu^2+^ species (Figure 5c), which is in agreement with literature that reports on the fluorescence quenching of indole derivatives by copper ions and a few other metals [28,34,35]. The Fe^3+^ exhibited a prominent fluorescence quenching, as previously observed for the fluorescent derivatives of dopamine used to quantify iron [36,37]. The fluorescence quenching was also large for Au^3+^ and Cr_2_O_7_^2−^ ions but associated with low data reproducibility, which is also due to the formation of gold nanoparticles. Based on these results, and considering that, to the best of our knowledge, there are no studies on copper ion detection exploiting the fluorescence quenching of a polymer derived by an endogenous molecule, such as SEDF, we decided to apply this method to real urine samples, where the presence of large amounts of copper ions would be indicative of a pathological condition (see below), whereas the concomitant presence of the other quenching ions, in particular Au^3+^ and Cr_2_O_7_^2−^, is insignificant in urine [38,39], and the urinary level of iron in healthy people (0.2 mg L^−1^, 0.003 mM) is approximately two orders of magnitude below the concentration of Fe^3+^ here tested (0.5 mM), and one order of magnitude below the minimum amount of Cu^2+^ here spiked in urine (0.05 mM, vide infra). However, there is also the possible interference of larger urinary levels, as a consequence of other iron-overload diseases, which could be simply minimized by using the proper sequestering buffer, as already reported [40,41]. Moreover, we also evaluated the interference of the main organic compounds in urine on SEDF fluorescence quenching. As shown in Figure 5d, urea, uric acid, creatinine, and citrate, in the physiological concentration ranges [42], did not lead to a fluorescence quenching. This was also true for glucose, hypoxanthine, glutamic acid, and ascorbic acid at the concentration that determines the pathological state. Accordingly, we proceeded to determine the Cu^2+^ concentration in buffer solution, artificial urine, and human urine samples.

### 3.3. Copper Quantification via Quenching-Based Bioanalytical Assay

**Figure 6:**
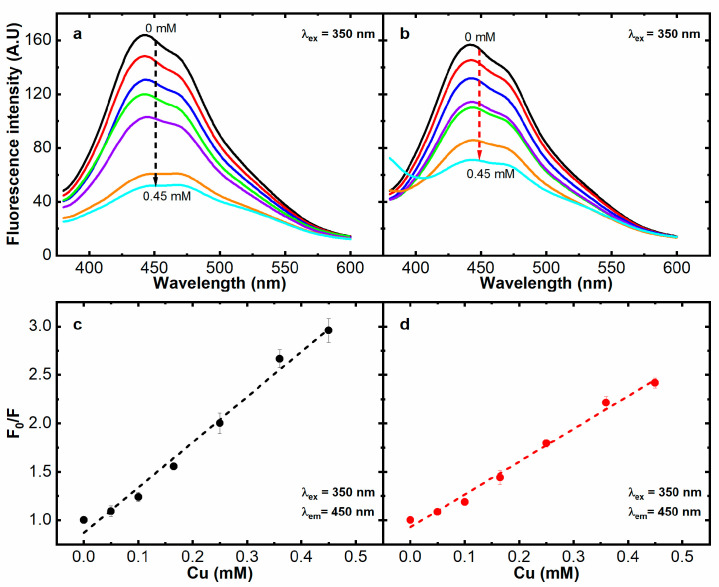
Fluorescence spectra and the relative calibration plots of the SEDF after copper ion addition in buffer (**a**,**c**) and in artificial urine samples (**b**,**d**). The fluorescence was recorded at 450 nm (λ_ex_ = 350 nm) and reported as a F_0_/F versus Cu^2+^ concentration (0, 0.010, 0.050, 0.100, 0.165, 0.250, 0.350, and 0.450 mM), where F_0_ and F represent the fluorescence intensity of the SEDF before and after the copper addition, respectively. The error bars represent the standard deviation (n = 4). The analytical parameters are reported in Table S1.

The SEDF fluorescence spectra and dose-response plots in the buffer and in artificial urine (AU) samples are reported in Figure 6. The fluorescence signal was reported as F_0_/F, where F_0_ is the fluorescence intensity of the SEDF, and F is the emission signal of the SEDF after the copper addition. The fluorescence intensity at 450 nm after the Cu^2+^ addition was plotted versus the Cu^2+^ concentration in the range of 0.05–0.5 mM, following a linear trend with remarkable analytical performance (Table S1), both in the buffer and in AU samples, as confirmed by the correlation coefficient *R*^2^ = 0.990 for both and by the linear fitting equations F_0_/F = 4.68 × [Cu^2+^] + 0.87 used for the Cu^2+^ detection in buffer and F_0_/F = 3.38 × [Cu^2+^] + 0.93 for AU. The excellent assay reproducibility was highlighted by the low variability (CV_av_% = 4% for buffer and 3% for AU), with an LOD of 16 ± 1 μg L^−1^ and an LOQ of 54 ± 2 μg L^−1^ in buffer solution and an LOD of 22 ± 1 μg L^−1^ and LOQ of 75 ± 3 μg L^−1^ in AU (Table S1). Notably, these concentrations are below the values of copper associated to the pathological state of Wilson’s diseases [24,25], stimulating the application of this assay to human urine samples.

### 3.4. Copper Detection in Human Urine Samples

With the aim to design a bioassay for the urinary Cu^2+^ quantification for clinical purposes, we transferred our experiments in ELISA-type 96-well microplates, allowing for the immediate and simultaneous analysis of several human urine samples and reducing the turnaround time for the analysis. Another advantage offered by the method here developed is the direct analysis of the urine samples without any pretreatment.

**Figure 7:**
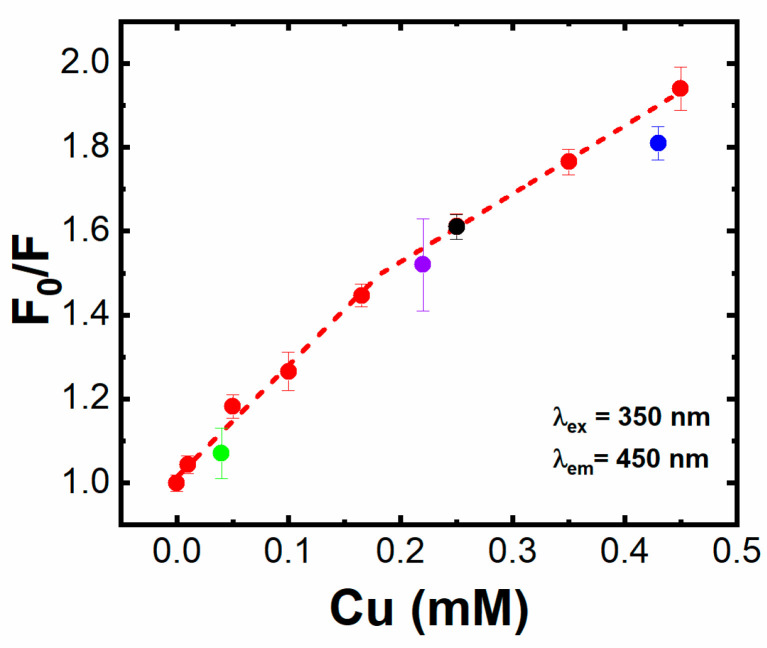
Calibration of Cu^2+^ in human urine (sample 3, red dots and fitting) and its quantification in the four urine samples, indicated by the different colors (1—green, 2—violet, 3—black, and 4—blue). The fluorescent signal is reported as F_0_/F at λ_em_ = 450 nm (λ_ex_ = 350 nm) versus the Cu^2+^ concentration, where F_0_ and F represent the fluorescence intensity of the SEDF before and after the copper addition, respectively. The error bars represent the standard deviation (n = 4). The analytical parameters of the calibration are reported in Table S2 and were obtained using the piecewise linear fitting implemented in the OriginPro software, version 2022. OriginLab Corporation, Northampton, MA, USA.

The urine samples were preliminarily assayed by ICP-MS to determine the starting copper physiological concentration, resulting in the nM range (Table S3). Among them, the urine sample n. 3 was selected to be used as a calibrator in the real matrix by Cu^2+^ standard additions (red line in Figure 7), since its Cu^2+^ level represents a mean value within the series. The CV_av_% (1%) and the slope reported in Table S3 highlight the high reproducibility and sensitivity of the method, even in the complex real matrix, with an LOD and LOQ of 59 ± 3 μg L^−1^ and 97 ± 11 μg L^−1^, respectively.

Subsequently, 100 μL of each urine sample was spiked with different known Cu^2+^ concentrations (0.05 mM; 0.20 mM, and 0.40 mM, see Table 1) and added to 1800 μL of the SEDF solution. These spiked samples were analyzed in parallel by fluorescence and ICP-MS (Table 1 and Figure 7).

Despite the complexity and the variability of the analyzed urine samples, the results clearly highlight the very good accuracy of the assay with respect to the calibrator, even if the urine sample n. 2 showed a slightly higher variability, likely due to the marked turbidity and the darker color of the sample. This allows us to foresee the realistic applicability of the method as a diagnostic tool, covering a broad range of Cu^2+^ concentrations expected in this type of patient.

### 3.5. Assay Performance Compared to Other Fluorescence-Based Method for Copper Detection

The quenching-based assay here designed was finally compared to the recently developed fluorescence-based methods for Cu^2+^ detection in urine [8,9,10,11] (Table S4), i.e., carbon dots (CDs) combined with covalent organic frameworks (COFs), chitosan/L-histidine-stabilized silicon nanoparticles (CS/L-His–SiNPs), nitrogen-doped quantum carbon dots (ND-CQDs), and blue CDs (bCDs) combined with green quantum dots (gQDs). These methods offer better LODs values [8,9] (except in Li et al. and Zhang et al., in which the LOD values were not calculated for human urine samples [10,11]). However, these nanostructures require long and laborious synthetic procedures. Contrarily, herein, SEDF was rapidly (2 h) synthesized and, for the first time, this novel biomaterial was applied for diagnostic purposes.

## 4. Conclusions

In this study, the fluorescent properties of a serotonin-derived fluorophore were successfully exploited for urinary Cu^2+^ determination via a quenching-based bioanalytical method. To the best of our knowledge, this is the first example of a quenching-based assay that exploits the fluorescent properties of a polymer derived by an endogenous molecule. The SEDF’s spectroscopic features were deeply investigated, and different techniques were applied to characterize this novel material, including mass spectrometry confirming its oligomeric state. The experimental conditions were optimized to obtain the highest emission signal and to improve the assay sensitivity and reproducibility. The whole protocol was performed within 2 h in line with clinical requirements. The Cu^2+^ ions were firstly quantified in buffer and in artificial urine obtaining very low variability, high sensitivity, and low LOD and LOQ values. Then, the assay was applied to estimate the Cu^2+^ concentration in human urine samples from volunteers and, finally, validated by ICP-MS obtaining very good recovery values and excellent analytical performances in a real matrix, namely, a CV_av_% of 1%, an LOD of 59 ± 3 μg L^−1^, and an LOQ of 97 ± 11 μg L^−1^. These values are below the concentrations of urinary Cu^2+^ that, for example, determine the pathological state of Wilson’s disease (60–240 μg L^−1^), enabling the applicability of the method for Cu^2+^ measurement in routine clinical practice without the need of sample pretreatment.

## Figures and Tables

**Table 1:** Cu^2+^ concentrations in the reinforced urine samples (Figure 7).

#	^1^ [Cu^2+^]	F_0_/F	^2^ [Cu^2+^]	^3^ [Cu^2+^]	^4^ Recovery (%)
1	0.05 mM	1.07 ± 0.06	0.04 mM ± 0.02	0.03 mM	133%
2	0.20 mM	1.52 ± 0.11	0.22 mM ± 0.01	0.21 mM	105%
3	0.20 mM	1.61 ± 0.03	0.25 mM ± 0.01	0.22 mM	114%
4	0.40 mM	1.81 ± 0.04	0.43 mM ± 0.01	0.49 mM	88%

^1^ Spiked; ^2^ quenching-based method; ^3^ ICP-MS; ^4^ 100 × [Cu^2+^]_Quenching_/[Cu^2+^]_ICP-MS_.

## Data Availability

Data available upon request.

## Supplementary Materials

The following supporting information can be downloaded at: https://www.mdpi.com/article/10.3390/s23063030/s1, Figure S1: Fluorescence stability over time for serotonin-derivative fluorophore (SEDF); Table S1: Cu^2+^ detection in buffer and in AU. Data are from Figure 6; Table S2: Cu^2+^ quantification in human urine. Data are from Figure 7; Table S3: Physiological Cu^2+^ concentration of human urine samples; Table S4: Fluorescent-based assays for Cu^2+^ quantitative analysis in human urine samples.
