# Supplementary material for: Serotonin-Derived Fluorophore: A Novel Fluorescent Biomaterial for Copper Detection in Urine
Source: Sensors (Basel). 2023 Mar 10;23(6):3030. doi: 10.3390/s23063030 (PMC10055690; doi:10.3390/s23063030)
Supplement: Supplementary file 1 [file sensors-23-03030-s001.zip › sensors-2286112-supplementary.pdf]

Supplementary Materials

Serotonin-Derived Fluorophore: A Novel Fluorescent Biomaterial for Copper Detection in Urine

Mariagrazia Lettieri [1], Simona Scarano [1], Laura Caponi [2], Andrea Bertolini [3], Alessandro Saba [2,3], Pasquale Palladino [1,*] and Maria Minunni [1,*]

[1] Department of Chemistry 'Ugo Schiff', University of Florence, 50019 Sesto Fiorentino, Italy
[2] Laboratory of Clinical Pathology, University Hospital of Pisa, 56126 Pisa, Italy
[3] Department of Surgical, Medical and Molecular Pathology and Critical Care Medicine, University of Pisa, 56126 Pisa, Italy
* Correspondence: pasquale.palladino@unifi.it (P.P.); maria.minunni@unifi.it (M.M.)

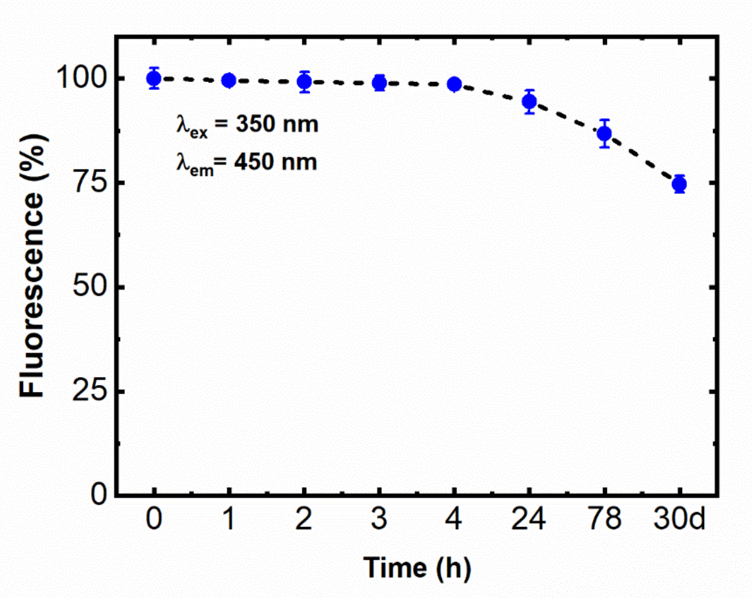

Figure S1. Fluorescence stability over time for serotonin-derivative fluorophore (SEDF). SEDF was obtained by heating 2 g L^{-1} serotonin at 60 °C for 2 h in 10 mM TRIS at pH 9.00. Fluorescence intensity was recorded at 0, 1, 2, 3, 4, 24, 78 h, and after 30 days (30d). The error bars represent the standard deviation (n = 4).

Table S1. Cu^{2+} detection in buffer and in AU. Data from **Figure 6**.

Matrix	Equation	m (mM)	LOD (μM)	LOQ (μM)	[1]LOD (μg L^{-1})	[1]LOQ (μg L^{-1})	R^2	CV_{av}%
Buffer	y = 4.7x + 0.9	4.7 ± 0.2	0.26 ± 0.01	0.85 ± 0.04	16.3 ± 0.7	54 ± 2	0.990	4
AU	y= 3.4x + 0.9	3.4 ± 0.1	0.35 ± 0.01	1.18 ± 0.05	22.6 ± 1.0	75 ± 3	0.990	3
[1] LODs and LOQs are reported in μg L^{-1} to compare them to the values of Cu^{2+} that determine the pathological state, *i.e.*, 60-240 μg L^{-1}.								

Table S2. Cu^{2+} quantification in human urine. Data from **Figure 7**.

Calibration curve: $F_0/F= 1+ k [Cu^{2+}]$								
Matrix	$[Cu^{2+}]$ (mM)	k (mM)	LOD (μM)	LOQ (μM)	[1]LOD (μg L^{-1})	[1]LOQ (μg L^{-1})	R^2	CV_{av}%
Urine	0 - 0.165	2.61 ± 0.15	0.93 ± 0.05	3.1 ± 0.2	59 ± 3	97 ± 11	0.997	1
	0.25 – 0.45	1.64 ± 0.14						
[1] LODs and LOQs are reported in μg L^{-1} to compare them to the values of Cu^{2+} that determine the pathological state, *i.e.*, 60-240 μg L^{-1}.								

Table S3. Physiological Cu^{2+} concentration of human urine samples.

Urine sample	[1]$[Cu^{2+}]$ (nM)
1	0.31
2	0.13
3	0.05
4	0.08
[1] $[Cu^{2+}]$ was determined via ICP-MS prior of copper addition.	

Table S4. Fluorescent-based assays for Cu^{2+} quantitative analysis in human urine samples.

Material	Fluorescent method	LOD (μg L^{-1})	LOQ (μg L^{-1})	Matrix	References
[1]SEDF	Quenching	59 ± 3	97 ± 11	Human urine	This work
[2]CDs@COFs	FRET	4.19×10^{-4}	-	Human urine	[8]
[3]CS/L-His–SiNPs	Quenching	3.49 ± 0.03	-	Human urine	[9]
[1] Serotonin-derived fluorophore; [2] Carbon dots (CDs) combined with covalent organic frameworks (COF)s. [3] Chitosan/L-histidine–stabilized silicon nanoparticles (CS/L-His–SiNPs).					
